# Government Information Dissemination During Public Health Emergencies: An Analysis of China's Experiences

**DOI:** 10.3389/fpubh.2022.748236

**Published:** 2022-03-22

**Authors:** Yuye Zhang, Jiahao Shan, Zheyou Ye

**Affiliations:** School of Journalism and Communication, Hubei University, Wuhan, China

**Keywords:** public health emergencies, COVID-19, information disclosure, empirical theory, government websites

## Abstract

Information disclosure is crucial in China's official response to the COVID-19 pandemic. Since the early phase of the pandemic, the government's method of communication has relied heavily upon its analysis of information disclosed during past public health emergencies. This approach was proposed to better inform and prepare citizens during the crisis. This study aimed to study the effectiveness of China's information disclosure by examining themes, interconnection, and timeliness of information as posted on the Weibo microblogging platform between January and April 2020. The Latent Dirichlet Allocation (LDA) topic model analysis for social networks revealed six main characteristics including a shift from 'scattered' to 'focused' communication. Three main themes surrounding experience were highlighted, namely social governance, medical expertise, and encouragement, although experiential knowledge disclosure was timelier than other topics. This study broadens the dimension and scope of empirical theory by examining government information disclosure practices and provides a reference for further research.

## Introduction

As of April 1, 2021, more than 130 million cases of COVID-19 were diagnosed worldwide. The gravity of the situation prompted António Guterres, the U.N. Secretary-General to describe the pandemic as “the biggest challenge the world has faced since World War II” (1). The international community's recognition that COVID-19 was more than China's domestic issue was useful in the sharing of data to facilitate timely production and disclosure of information. The nature of the pandemic underscored the importance of good communication to alleviate panic and prioritize notification of procedures for good health care. To this end, the Chinese government undertook to release information to the public as a means of engaging and responding to citizen's concerns. Multiple channels of dissemination were used, including government press conferences, advisories from health experts, news reports, and public comments. Overall, the information disclosed correlated with the country's awareness of the disease, the rate of spread, citizen's perception of risk (panic and dismissal), and the level of safety. Moreover, information dissemination was closely aligned with the government's preventative strategies against this infectious disease.

Much of the information disseminated and decision-making by the Chinese government regarding the treatment of the disease was based on experiential knowledge. Given China's early reticence at the initial outbreak in Wuhan, it was not surprising that further handling of the pandemic narrative came under global scrutiny. Specifically, scholars and governments were interested in how information between the Chinese government and its citizens were being handled, and the latter's acceptance of that information. The purpose of this study, therefore, is to contribute to the scholarly literature by investigating the scope and extent of China's communication with its citizens during the pandemic. The research identifies the main types of information disclosed at the initial stages, China's relationship with the population, the timeliness of dispensed information, and its effects on the public if any.

On May 7, 2003, the 7th Executive Meeting of the State Council endorsed the definition of a public health emergency proposed by the Regulations on Public Health Emergencies committee. Public health emergencies refer to outbreaks of major infectious diseases of epic proportion including idiopathic diseases, foodborne diseases, occupational poisonings, and other contagions. These afflictions all occur suddenly and pose a serious challenge to public health. As a public health emergency, COVID-19 unearthed gaps in China's operational procedures. Many major agencies had to be remodeled and efforts were made to reduce the knowledge gap between citizens. To manage hospitalization and gain control over the high caseload of infected persons, it was necessary to prioritize the protection of medical personnel. Accordingly, there was the need to insist on handwashing hygiene and isolate or quarantine sources of infection to prevent widespread harm to the public (2). COVID-19 also proved to be disruptive to other sectors such as education. Fortunately, technology could be optimized to facilitate the switch to online education, albeit the network environment and hardware equipment needed to be integrated with online learning platforms and learning management systems. This meant that resources were needed to provide high-quality instruction and improve the legal and regulatory systems (3). The job market was also affected since the implementation of policies and regulations for the resumption of work was essential to strengthen multi-party incentives. These strategies had to consider the role of emergency response, coordinate resources, guarantee basic capacity and capabilities. In some cases it was necessary to revamp various policy tools, rationally schedule measures and policies, and extend policy enforcement (4).

The dissemination of government information was found to be significant for the management of various public health emergencies, according to recent studies in emergency management (5). Open government information was paramount for COVID-19 since stakeholders needed to adopt efficient public policies to mitigate losses and reduce economic and social costs.

### China's Response to Reform

After some 25 years of uneven progress toward greater government transparency, China adopted its Open Government Information Regulations (hereafter referred to as “The Regulation”) on May 1, 2008. One specific administrative agency was assigned the responsibility to maintain and update all aspects of government information disclosure. The agency had five specific functions: (1) organize the compilation of guidelines to disclose information, (2) catalog government information such as annual reports, (3) incorporate organizational information, (4) review the information to be disclosed, and (5) any other duties related to disclosure.

China's experience with Severe Acute Respiratory Syndrome (SARS) aided its ability to respond to public health emergencies (6). When looked at holistically, public health emergencies are closely associated with scientific methods and expertise in the management of public information, public opinion, public relations, and public image (7). Since COVID-19 occurred at a time of ubiquitous information technology, scholars were able to gain new insights into the construction of shared information. Rapid sharing of scientific data during public health emergencies serves to mitigate public panic (8). After clarifying legal boundaries about what can be disclosed, information such as media releases can provide insight into areas such as social psychology (9).

The Regulation was highly congruent with the government's need to focus on the public's needs (10, 11). As a next step, the government classified citizen's needs to determine the best way to disclose relevant information (12), to effectively meet needs. This information-sharing model was useful to bridge situations in which frail and vulnerable individuals had difficulty accessing medical assistance due to the information gap (13). The model was able to deal with other factors such as online public opinion generation, early detection and warnings, mid-term processing, guidance, recovery, and reflection.

This research attempted to clarify the importance of information disclosure in managing public health emergencies. We propose that when information is mixed with rumors and vague reports that are difficult for ordinary people to discern, it needs to be analyzed and clarified by authorities and experts (14). China's information disclosure reasoning was based primarily on its experience with public health emergencies. Therefore, experience played an important role in the management of the COVID-19 pandemic, especially for disclosed information.

## Theoretical Framework

This paper explores information disclosure in China's response to COVID-19 using information theory. Information theory can be traced to Claude Elwood Shannon, who improved upon the original concept proposed by H. Nyquist and R.V. L. Hartley, in a 1948 paper entitled “The Mathematical Theory of Communication.” According to the theory, anything that reduces uncertainty in a situation is information, which is a way, form, or movement of matter attributed to a universe of things, generally known as data. In moments of crisis, information can help alleviate uncertainty (15).

A better understanding of the meaning and significance of experience in information disclosure is consequential to the theory. In ancient Greece, doctors used empirical methods to diagnose and treat patients. Today, it usually refers to knowledge gained through experiments or research, as opposed to intuition and superstition. However, experience is sometimes the only way to acquire and release life's secrets, and within this context, the way natural events are revealed (user experience in natural sciences), is deepened and enriched. According to Dewey, this philosophy of nature also guides the further growth of experience, so this process of change may accelerate (16).

The theory of empiricism has expanded in these modern times since the 1600s. Pragmatic empiricism is derived from Dewey's emphasis on the unity of theory and practice, the use of subjective human initiative, and opposition to blind retention in negative thinking (17). Correspondingly, the methods and processes of mankind's transformation in nature indicate the significance of the experience to social progress and the enrichment of experience through the process itself (18).

SARS and COVID-19 are public health emergencies that endanger the safety of the public in a relatively short period while causing great damage to society. While governments and citizens cannot predict and prepare specifically for infectious diseases (19), experience is one way of testing timely responses to these events.

Most of the research on public health emergencies in China result from experiences learned during the SARS interval. This experience encompasses social governance learned through studying the emergency response mechanism of western developed countries to public health emergencies (20); medical collaboration such as sharing the diagnosis and clinical treatment (21); managing news propaganda such as staged and focused propaganda, and understanding the direction of public opinion (22).

Scholars generally approach the study of public health emergencies as a macro perspective of social governance. This concept often posits four categories of experiences. The first requirement is to improve the system for emergency management through integration, systematization, specialization, coordination, and standardization in public health emergencies (23). The second is to establish and improve the organization of emergency management. The third is to improve the emergency management system by establishing monitoring, warning, and response systems that focus on prevention (24). The fourth is to align the fight against major epidemics to build the party's governance capacity, enhance urban governance, improve people's health, and revive Mainland China (25).

These studies are evidence that detailed investigations and summaries of diverse experiences regarding the epidemic pre- and post-SARS were centered mainly on the macro-level relationships between public health emergencies and experience. Thus, a gap exists between experience-based information disclosures at the micro-level in public health emergencies.

Information disclosure is a form of data dissemination. Experience sharing is an essential mechanism of dissemination so that the organism becomes a social and individual being that builds a community (26). During COVID-19, the Chinese government, medical experts, media, and the general public disclosed information that was redeeming and enabled social stability (27). Moreover, the Chinese government's microblogging website, Weibo, became the preferred method for citizens to communicate and evolved into an analytical form of citizens' knowledge and expression (28).

Consequently, Weibo was used to summarize citizen's shared experiences during public health emergencies by analyzing microblogs on the platform. The process involved a discussion of the categories of government's experience-based information. This includes the relationship between disclosure and dissemination, and its timeliness. Weibo also provided insight and suggestions for improving and optimizing the dissemination of public health information in the future. Overall, it was possible to broaden the interpretation dimension and the empirical theory of disclosure practices for government information along with their practical and theoretical significance.

As a result, the main research questions of this paper are as follows:

Research question 1: What are the types of experience required for disclosure of government information during COVID-19?

Research question 2: What is the connection between experience and government information disclosure during COVID-19?

Research question 3: Is the government's information disclosure practices based on timely experience during COVID-19?

## Methods

This study used Python software to crawl 13 timeframes of Chinese-language messages posted on the Weibo microblogging platform between January 11 and April 8, 2020 (see Table 1). According to the Wuhan Municipal Health Commission, January 11 was a significant date because it was the first time the name of the virus was changed from “unexplained pneumonia” to “new coronary viral-infected pneumonia.” Additionally, it was the first time the Wuhan municipal revealed information on COVID-19. Another important date was the reopening of Wuhan City on April 8. The 11 timeframes between these dates revealed the government's response to COVID-19.

**Table 1:** Inclusive timeframes of news events during COVID-19.

**Time**	**Important Events**
January 11	Wuhan municipal health commission pioneered the name change from “unexplained pneumonia” to the “new coronary virus pneumonia.”
January 20	Zhong Nanshan made it clear that the virus has a “human-to-human transmission” infectivity.
January 22	Hubei initiated a secondary emergency response to public health emergencies.
January 23	Wuhan was shut down at 10 a.m. Zhejiang, Guangdong, and Hunan initiated the first-level response to major public health emergencies. Construction of Vulcan Mountain Hospital began.
January 27	The state council's joint prevention and control mechanism comprehensive team hold a press conference to notify 31 provinces (autonomous regions, municipalities) and Xinjiang Production and Construction Corps. to report various cases, including newly diagnosed, newly cured, new severely ill, recently died cases, etc.
January 29	Hubei Province red cross announced the use of the first batch of donated materials for the prevention and control of the new corona pneumonia.
Feb 1	Hubei Province extended the 2020 Spring Festival holiday.
Feb 7	The ministry of education stated that no one was allowed to return to school without approval. Shenzhen, Guangzhou, and Chengdu implemented community closures.
Feb 19	The ministry of human resources and social security expanded the scope of unemployment insurance.
Mar 3	Zhejiang Province released information on seven (7) newly confirmed imported overseas cases.
Mar 19	The investigation team of the State supervision commission issued the “Notice on the investigation of the situation” which involved Dr. Li Wenliang's reflection by the masses.
Mar 30	The central group leading the response to the new corona pneumonia epidemic required that all localities adhere to the open and transparent release of information and did not allow under-reporting or over-reporting in pursuit of zero cases.
April 8	Wuhan re-opened.

### Sample

We chose the study sample for three reasons. First, it was important to examine the content of government information disclosed during the period to understand the government's attitude and handling strategies of the COVID-19 public health emergency. By following the microblogs of local governments during the period, we examined the content of government information disclosure to assess the effectiveness and/or limitations of government governance. Second, government information disclosure was premised to reduce the public's state of panic and anxiety due to the complexity of the epidemic. Third, since COVID-19 was a new major health event, the government needed to draw on its experience to make judgments and major decisions. From January 11 to April 8, the microblogs had extensive information about the government's experience handling public emergencies. This was useful insight about government information disclosure based on experience. Since government information was mainly transmitted to online media instead of the traditional media during the initial stages of the epidemic, we chose to use mainstream government microblog accounts as the data source.

## Procedure

We inputted the phrase “new coronary pneumonia” into Weibo and filtered the data by the specific date range. After harvesting a total of 135,921 entries, excluding invalid data reduced this number to 45,327. We then selected the top 20 microblog accounts since the research objective was primarily to use current experiences during the outbreak. As shown in Table 2, these accounts had the most information releases and were useful as sample data for the research.

**Table 2:** Top 20 government media Weibo accounts with the highest number of information releases.

**Account ID**	**Administrative unit of account holders**	**Quantity (items)**
Voice of Zhejiang	Zhejiang people's broadcasting station	237
Fm1036 of Fujian news broadcast	Fujian people's broadcasting station	215
Emergency Broadcasting in Hubei Province	Hubei radio and television station	202
People's daily online	Central committee of the communist party of China	193
Our video of Beijing news	Beijing municipal committee of the communist party of China	137
Published by Xiangshan	Xiangshan county committee of the communist party of China	129
Jilin traffic radio	Jilin radio and television station	107
China news network	China news service	96
China news weekly	Overseas Chinese affairs office of the state council of the communist party of China	84
Daily economic news	Chengdu media group	81
China news service	General information administration of the communist party of China	75
Justice Nankang	People's procuratorate of nankang district, Ganzhou City	69
Western net	The department of publicity of the CPC Shaanxi provincial committee	64
Global net	People's daily, an organ of the central committee of the communist party of China	62
Rain City TV	Ya'an broadcasting and television station	58
Xin'an evening news	Anhui daily newspaper group	57
China family news	Population and cultural development center of national health commission	56
Published by Zongyang	People's government of zongyang county, tongling city, Anhui Province	54
Yangcheng evening news	Yangcheng evening newspaper group	53
Daily business news	Hangzhou daily newspaper group	52

COVID-19 was China's most severe public health emergency after SARS. To compare the data between the two, we collected and sorted the government's main response strategies during the SARS outbreak, and information on various measures, then analyzed the keywords as shown in Table 3. This step helped to organize the empirical information on COVID-19.

**Table 3:** China's main response strategies and keywords for SARS.

**Keywords**	**Government's response strategy during SARS**
Pay taxes, deferment, taxation, policy	The Beijing municipal local taxation bureau handled the policy of deferring personal tax payments during the SARS epidemic.
Coupons	During the 2008 global economic and financial crisis, Chengdu Civil Affairs Bureau issued 37.91 million Yuan in coupons.
Prevention and control command	SARS prevention and control headquarters were established throughout the country.
Ministry of health, notification, and data	The ministry of health reported national data on SARS daily.
Airport, investigation, isolation, and immigration	The capital airport implemented screening and quarantine measures for the entry and exit of personnel.
Appointment, masks, protective clothing, production, designated hospitals	The government-appointed Changshan group and Minglu Garment Group as designated manufacturers of masks and protective clothing. The Ministry of Health announced designated hospitals for treatment.
Social solicitation, device, equipment	The capital museum and the Hebei provincial museum solicited medical equipment and protective equipment from the community.
Live broadcast, press conference	Beijing TV station broadcasted live streams of SARS press conferences.
Frontline, public security, civilian police	A total of 1.7 million police officers participated in the front-line fight against SARS.
Enrollment expansion, postponement of exams, online teaching	For students affected by the SARS epidemic, the government enacted enrollment expansion for graduate students and implemented policies such as the postponement of exams for grades 4 and 6. In addition, Tsinghua University opened online portals to conduct non-contact teaching.
Community, closed management, health certificate	The Beijing community was closed to manage infection rates. The general administration of quality supervision, inspection, and quarantine required entry and exit personnel to apply for physical signs and health certificates during the SARS period.
Free, refund, tracking, and contacts	The Civil Aviation Administration of China (CAAC) announced that student refunds will be exempted from handling fees from May 1st. The ministry of health required the tracing of contacts of all SARS cases.
Close venues and large-scale events	The government required all large venues to be closed and large events to be canceled.
Publicity	Nationwide publicity activities were carried out to prevent the spread of SARS.
Dismissal	The dismissal of 10 cadres was announced in Longnan County, Ganzhou City, and Jiangxi Province.
Zhong Nanshan, interview	Zhong Nanshan accepted an invitation for an interview with CCTV reporters to relieve the public's anxiety about SARS.
Volunteer	Local public security, health, epidemic prevention, and community youth formed a volunteer service group for SARS prevention.
Government, emergency plan	Local governments formulated emergency plans for SARS prevention and work control.
Vegetables, materials	To assist the shortage of vegetables in Beijing, Hebei coordinated emergency food and supplies to support Beijing.
Pass	The ministry of transport of the People's Republic of China issued an emergency notice of the special pass for the transportation of emergency materials against SARS.
Medical insurance	Medical insurance offices and health institutions included SARS in the municipal medical insurance list.
The first case, confirmed, imported, cured	The media reported on the first confirmed SARS patient, the first imported SARS patient, and the first cured patient.
Medical expert, wear, disinfectant, mask	Medical experts and the government requested all persons to wear masks and use disinfectants.
Medical staff, support	National medical staff supported Beijing's fight against SARS.
SARS ward	Academician Wang Chen, the leader of the SARS expert group, established the SARS ward.
Xiaotangshan Hospital designated ward	The government constructed Beijing Xiaotangshan Hospital as a designated ward for SARS.
Throat swabs, research and development, nucleic acid testing, kits	Throat swabs were collected from SARS patients and nucleic acid detection kits were developed for the diagnosis of SARS.
Treatment plan, classification treatment	The ministry of health announced the recommended treatment plan for SARS. These were divided into seven types of treatment plans.
Chinese Medicine	All 36 SARS patients admitted to The First Affiliated Hospital of Guangzhou University of traditional Chinese Medicine were cured through the treatment.
Patients, reports	The media reported on the anti-epidemic process for SARS patients.
gratitude	The media reported that the Anhui Provincial Women's Federation and the Tonight News thanked the frontline medical staff workers of SARS.
Donate blood	The media reported that Shanghai Blood Donation Office called on citizens to donate blood.
Party member	The media reported on the pioneering and exemplary behavior of party members in the fight against SARS.

Overall, 135,921 pieces of information were retrieved manually based on keyword searches. Using LDA-based topic modeling to discover the topics in the social media content, we extracted data irrelevant to the research sample. As a result, 65,453 Weibo keywords were selected for the multidimensional analysis of network and timeliness.

To address potential ommissions from the existing corpus, we analyzed reports and literature on related public health events about SARS, the avian influenza A (H7N9), and COVID-19. The new word divisions were sorted and added to the database for word segmentation generating terms such as “square cabin,” “new coronary pneumonia,” “lockdown,” and other terms which were used to capture and divide the various types of experience-based information.

We then merged synonyms within the research objective. For example, “new coronary pneumonia” and “novel coronary pneumonia” became “new coronary pneumonia.” Similarly, the terms “China” and “People's Republic of China” became “China.” Last but not least, we replaced some keywords, such as “Xiaotangshan Hospital” with “Leishenshan Hospital” and “Huoshenshan Hospital,” and “Health Certificate” with “Health Code” and “Social Welfare.”

## Results and Discussion

The research on information disclosure extends beyond Weibo microblogs. Chinese scholars such as Lv Wenbao have examined the subject using similar dimensions albeit on alternative media platforms. Other researchers have examined information disclosure on mainstream media and the shakeout among online video platforms such as People's Daily. These platforms focused on issues such as China's assistance to the international community and evaluated the government's progress, response time, and notifications during the epidemic (29). Huang and others analyzed government information disclosure on TV channels, newspapers, and other media channels (30). Their research showed that central-level government media concentrated more on topics like public crises and social governance, while local government media tended to formulate positive and more optimistic content (30). An examination of the research questions showed the sample included the content of government information disclosed by mainstream media across major traditional and new media platforms. The findings of this study are posited to have wider interpretive powers in concurrence with scholars like Lv Wenbao and Huang Zaobin.

### Analysis of LDA Topic Modeling

The analysis of 65,453 microblogs using the TF-IDF algorithm of the LDA-based model of the government's experience-based information disclosure text (31), introduced 10 core topics to the corpus. A topic model is a machine learning clustering algorithm based on probabilistic statistical techniques to extract and summarize topics and identify potential clusters from large text data or corpora. This is a three-layer Bayesian model based on “text-topic-vocabulary,” which is automatically identified and categorized by a computer algorithm. Table 4 shows the eight keywords for each topic. We then repeated the combination of these 10 topics and identified three main themes: Experiences in social governance, medical experience, and encouraging support.

**Table 4:** Subject classification of disclosure of experience information.

**Topics**	**Keywords**
Topic 1	COVID-19, epidemic, companies, measures, policies, guarantees, banks, consumer vouchers
Topic 2	Anti-epidemic, triumph, love, new coronary pneumonia, quarantine, medical staff, refueling, discharge
Topic 3	Epidemic, isolation, COVID-19, support, action, medical team, Wuhan, Chinese medicine
Topic 4	News, COVID-19, live broadcast, epidemic, press conference, epidemic, security, isolation
Topic 5	COVID-19, epidemic, disinfection, masks, support, nurses, shelter, testing
Topic 6	Wuhan, people, encouragement, thank you, party members, COVID-19, cure, frontline
Topic 7	Nurses, doctors, isolation, fever, breathing, vaccines, testing, Vulcan Mountain
Topic 8	COVID-19, epidemic, Wuhan, community, security, information, joint prevention, and control, platform
Topic 9	Epidemic, Wuhan, security, tracking, quarantine, new addition, volunteers, confirmation
Topic 10	COVID-19, epidemic, isolation, first case, Wuhan, guarantee, emergency plan, measures

Experiences in social governance (47,486 items) consisted of subthemes 1, 4, 8, 9, and 10, which accounted for 72.55% of the total. The data revealed several aspects of society were affected by the pandemic such as policy, epidemic information, economics, education, and livelihoods. The trend of the epidemic was guided because of this experience. Second, medical experience (12,324 items) contained subthemes 3, 5, and 7, which accounted for 18.83% of the total. Epidemic prevention guidance, patient treatment, vaccine research, and inauguration were discussed. Professionalism played a key role in saving citizens' lives and preventing the further spread of the epidemic. The third was the inspiring and motivational theme comprised of subthemes 2 and 6 (5,642 items), accounting for 8.62% of the total. This included reports on the attention and dedication paid to patients, medical workers, and people from all walks of life during COVID-19. Having this experience played a pivotal role in maintaining social harmony and stability, motivating patients, and building a sense of camaraderie.

### Results of Social Network Analysis

**Figure 1:**
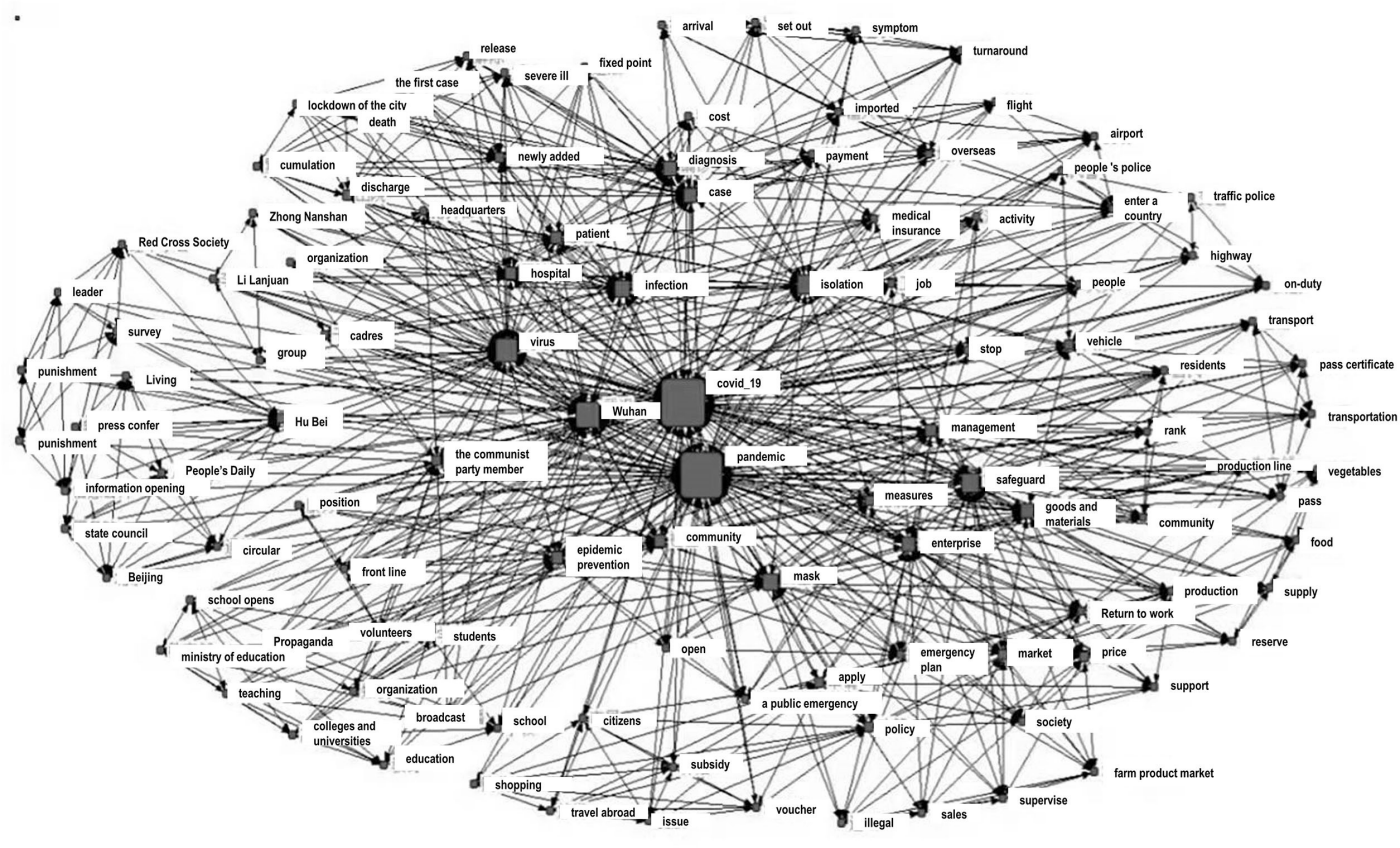
Social network analysis of experience information disclosure.

Researchers mined 65,453 microblogs for high-frequency words and used social network analysis to understand the internal connections to information sharing in the future (see Figure 1). By comparing multiple indicators such as degree centrality, closeness centrality, betweenness centrality, and eigencentrality (32) in the social network graph, the core node identified COVID-19 and the epidemic in the network analysis. Primarily, the nodes showed how government information disclosed during the epidemic focused on the event itself and gave a clear impression of social governance. Second, terms such as “isolation,” “security,” and “Wuhan” emphasized the need for social governance. Lastly, keywords about the content of information disclosure showed partial collaborative relationships such as the State Council, Beijing, and press conferences; passes, traffic, transportation; subsidies, consumer vouchers, citizens, and tourism. Many of these keywords appeared simultaneously and were closely related, indicating that many social actors were involved in information disclosures.

### Statistic Results for Release Time of Information Disclosure

The researchers conducted statistical tests on the release time of the three types of experiences from the topic modeling analysis. They selected five information disclosure contents for each category and calculated the time when the information was disclosed (7). In the extant literature on the timeliness of information disclosure during public emergencies, Zhang and Wang (33) confirmed that 36 h after an outbreak, netizens were extremely alert. Thus, information disclosure within this period can be considered as having high information dissemination value and support the timeliness characteristic of information disclosure.

Table 5 presents the three types of experience-based information. Generally, the disclosure time was within one or several hours, and none of them exceeded 24 h. The official WeChat public of the National Medical Insurance Bureau, for example, announced a “special reimbursement policy” for patients diagnosed with pneumonia on January 21. The World Wide Web announced this information at 8:16 p.m. on the Weibo platform based on the information disclosed about medical experience. Since scholar Zhong Nanshan first announced the person-to-person transmission of the new coronary pneumonia on January 20, citizens nationwide began monitoring the progress of the new disease and commenting about the increase in the number of diagnosed patients. A day later, the Xin'an Evening News reported that the Anhui Traditional Chinese Medicine Association had prescribed the use of traditional Chinese medicine for new coronary pneumonia cases. The January 20 disclosure signaled a turning point in the determination of human-to-human transmission. During the following days, reports were posted about heroic deeds by medical staff; 2 days later, altruism by ordinary people and party members was reported as well. In light of the improvement in the condition of hospitalized patients at the beginning of January, the media posted messages of encouragement to persons diagnosed with the disease. Disclosing encouraging information was relatively timely and conformed to the established rule of reporting public health emergencies to front-line staff and patients.

**Table 5:** Statistical results of the disclosure time of experience information.

**Information disclosure content**	**Weibo account**	**Release time**
**Experience in social governance**		
Experts announced that COVID-19 indeed spread from person to person.	China news weekly	19:29 Jan 20
Treatment costs for patients diagnosed with COVID-19 began to be covered by medical insurance.	Global net	20:16 Jan 21
Zhejiang initiated the first-level response to major public health emergencies.	People daily net	16:11 Jan 23
Solicit medical substances from society.	China news network	15:59 Jan 24
Huzhou issued policies to support enterprises and resume work in response to the epidemic.	Voice of Zhejiang	18:18 Feb 3
**Medical experience**		
Experts reminded citizens of epidemic prevention practices	Voice of Zhejiang	13:03 Jan 20
Anhui Provincial Society of Traditional Chinese Medicine issues Chinese medicine prescriptions for COVID-19.	Xin'an evening news	20:52 Jan 22
Medical staff across the country began to support Wuhan.	Voice of Zhejiang	12:10 Jan 24
Wuhan announced the start of the construction of Huoshenshan hospital.	People's daily online	19:39 Jan 24
Wuhan built Square Cabin hospital.	Daily economic news	23:18 Feb 3
**Encouragement experience**		
Report on the dedication of the medical staff.	Daily business news	10:01 Jan 21
Report on the contributions of party members and ordinary people.	Daily business news	14:40 Jan 23
Report news that inspires patients.	Western net	10:25 Jan 31

Based on their experience with social governance, the central government media (Global Network, People's Daily, China News Weekly, and China News Network) paid more attention to information disclosure. Meanwhile, local government media (Zhejiang Voice, Xin'an Evening News, and Daily Economic News) focused on medical experience, so this information was disclosed relatively quickly albeit more attention was given to the disclosure of encouraging information (Daily Business, Western Network). As this type of information usually requires some post-production, and materials must be obtained based on the spread of the epidemic, time sensitivity was relatively low. Yet information disclosure based on encouragement and the encouraging experience was relatively timely based on the escalating rates of the disease.

### Analysis of the Use of Empirical Information by Government Information Disclosure in Public Health Emergencies

#### From Peripheral to Focused

In his book, “A Mathematical Theory of Communication,” Shannon suggested that an exploration of information entropy brought people closer to the essence and core of information (15). Thus, information entropy is a quantitative measure of information uncertainty. To achieve better results in the dissemination of information in the wake of sudden public events, the uncertainty content must be reduced. Focused coverage of events strengthens attention to information from another perspective and helps construct information authenticity.

Al Ries and Jack Trout identified “The Law of Focus” as a key concept of marketing (34). Based on its tenets, the focus should also be placed on information dissemination since decentralized information cannot lead to a high diffusion effect (35). The results of the topic modeling analysis revealed that the government's experience-based information content disclosed during the COVID-19 pandemic was relatively extensive. Generally speaking, it can be divided into three categories: social governance, medical information, and encouraging support. While all three experience-based content of information disclosure differed, they all focused on fighting the epidemic.

The term “focus” reflects Chinese government department's more consistent media governance attributes in addressing public health emergencies and conforms to the narrative path of government media. In the SARS era, information disclosure was obscure (36) and information transmission lacked transparency, which weakened the social governance effectiveness of government media. During the COVID-19 phase, the three types of empirical information performed as expected and maintained a dynamic balance between dispersion and focus.

The disclosure of social governance information covered both macro and micro issues. These included the resumption of work orders and production policies, deployment of social resources, dissemination of epidemic information, and citizens advisory to prevent the spread of the disease. The dissemination of medical information concerned mostly patients' treatment, the transmission of epidemic prevention methods, the formulation of epidemic prevention strategies, and medical research. Encouraging support provided spiritual strength and reinforced the narrative for micro-groups, such as patients, medical workers, the masses, and party members.

Overall, the government's media information disclosure showed a clear focus on the effective contribution of government media to society, leading to good governance.

#### From Independence to Continuity and Interaction

The philosopher Dewey theorized the progress and formation of human beings were the results of working together under the principle of continuity of experience and interaction (37). China organically connected information disclosure experiences that originated at different moments, reorganized the isolated experience, and ultimately contributed to the effectiveness of government information disclosure during COVID-19.

We identified large amounts of information in the disclosure of government information under COVID-19 based on the government's main response strategies and various measures implemented during the SARS phase. This path reflects the continuity of experiences that were not identical but improved upon based on current conditions. For instance, during the fight against SARS, Beijing held a press conference and Beijing TV aired a live broadcast. Conversely, with COVID-19, news conferences were held nationwide, and CCTV and local TV stations provided live coverage. These responses were implemented based on the study of past SARS experiences and demonstrated the continuity of experience and improvements in the current processes. This illustrates the interactive nature of experience.

Public health emergencies are sudden and specific, and it is often impossible to immediately formulate the most effective response. However, using experience to formulate strategies and disclose information can often reduce casualties and losses. The Chinese government should actively epitomize and learn from all experiences in public health emergencies. These experiences should then be treated from a developmental perspective-combine current emergencies, discover deficiencies, and summarize new experiences to achieve continuous and interactive experiences. In this way, information disclosure based on experience can be more effective in the future.

#### From Presumption to Critical Thinking Based on Data-Based Context

Neil Postman's in-depth research on information control presupposed technology and tools can influence the form and structure of information. Consequently, human perceptions and values, based on the current digital media field, present a new form of data and media combination.

In her book “The Fate of Knowledge” Longino (38) suggested that critical contextual empiricism should be grounded in empirical data. Speculation and hypothesis are subjective, while data are objective and credible. To find the essence of better things, it is more analytical to distinguish the authenticity of things based on empirical data. This is evident in China's lack of experience in responding to public health emergencies during the SARS outbreak which resulted in delays in information disclosure. The presumption of informal epidemic control had several drawbacks and was reportedly ineffective.

In the early stages of COVID-19, China had not implemented systems as countermeasures to verify the travel trajectory of residents, which increased the difficulty of controlling the epidemic to a certain extent. To evade government regulations and limit human movement, some citizens falsely reported visits to high-risk areas. After March 7th, China relied on big data technology to help determine citizen's health status and monitor travels to potentially high-risk areas. The use of the “green health codes,” emphasized the digital trend of information disclosure based on existing experience. This illustrates how data-based empirical information was more critical than subjective judgment.

Moreover, it was reasonable for China to use data-based evaluative thinking on new media platforms to publish quotidian epidemic data and updates based on truths. From an evaluative perspective, government media should establish effective methods of information disclosure by abandoning untimely methods used in the past to avoid subjective and superficial disclosures, as they could result in arbitrary and incorrect judgments. This concretizes China's information disclosure mechanism for responding to public health emergencies.

#### From Spirit to the Combination of Spirit and Body

Among Western philosophers, the “I think” model has been supported by many scholars regarding empiricism. Immanuel Kant, Plato, and Parmenides of Elea supported the “I think” viewpoint and theorized knowledge came from rational thinking and had no connection with experience and feeling. Kant believed that “because the sensory world places such strict limits on intellectuality, Plato abandoned it and bravely raised the wings of the idea to fly to the other side of the sensory world and enter the vacuum of pure intellectuality” (39). Correspondingly, Plato believed that perception cannot be used to obtain knowledge, but reason should be used to make judgments. Their empiricism emphasized that knowledge should come from rational thinking and judgment, and experience gained through the senses cannot be called knowledge. This is an inappropriate argument in this context. First, the results from the social network analysis showed information disclosure content contained perceptual and rational levels. Keywords such as “COVID-19” and the progress of the epidemic demonstrated the nature of notification and reporting. Both are strong perceptual information and play a vital role in stabilizing society.

These core contents are closely connected with the rational information provided at the implementation level of isolation, security, Wuhan, measures, etc., to achieve the effect of equal emphasis on social communication and social governance.

The German philosopher, Nietzsche, believed that sensation and reason were categorically fragmented as if they were two completely separate abilities. As a result, he completely shattered the reason itself and contributed to the incorrect separation of spirit and body (40). Experience should be a combination of sensibility and reason and should complement consciousness and practice. The same should be true of information disclosure based on experience. Therefore, in public health emergencies, the government's experience-based information disclosure should not only focus on the transmission of government and public information but also on adopting specific anti-epidemic measures, integrating macro decision-making and micro-practices for perceptual experience and rationality. The coupling of experience helps to enhance the value of information disclosure based on existing experience in public health emergencies, and better promotes the improvement of China's anti-epidemic capabilities.

#### Emulate to Screen and Recreate Use

SARS and COVID-19 are both public health emergencies, albeit in different eras. Consequently, the geographical spread, technical environment, and other external factors differ significantly. Using experience and information in government information disclosure is an imperative requirement in the new environment, which should be screened and verified.

During the SARS outbreak, traditional mass media such as television and radio were the main sources of government disclosures and dissemination of information. In contrast, during the COVID-19 epidemic, media integration and multi-channel dissemination were used more often. However, the experience of information disclosure is no longer emulative and attributive, but rather about recreating use after screening. Due to the difference in the virus itself, it is impossible to directly match the vaccine against SARS. However, it is necessary to develop a new vaccine based on past medical experience. Information disclosure in public health emergencies should take into account changes in the external environment and combine existing experience and current facts to establish a path for attribution to screening to new uses. This offers new value for China's information disclosure work in the future.

### Limitations of the Study

This study had several methodological shortcomings. First, the use of a series of time points during the pandemic as the basis for selecting government media Weibo accounts narrowed the results to a specific situation. Since it was impossible to assess all highly active media accounts during this period, the data is not reflective of all Weibo accounts and therefore not generalizable. However, the Weibo platform helped provide an overview of information disclosure based on experience.

Second, this study focused on the empirical-based information disclosure content of government media microblogging platforms. It analyzed the empirical-based information disclosure content of market-based media and offline media during the 19th National Congress of the Communist Party of China. Nevertheless, it is worth noting that this study is broadly consistent with existing studies on media channels such as shakeout platforms and newspapers, which indicate the practicality and validity of using microblogging platforms as a source of data collection. The research also reveals the heterogeneity of explanatory frameworks between different media platforms and channels due to differences in presentation forms and content generation methods.

Last but not least, the microblog data was analyzed to determine the effect of government media's experience-based information disclosure content. By using questionnaires or telephone surveys, future research should strive to better understand the specific impact of information disclosure on citizens. Further research will also be needed to demonstrate the impact of empirical information disclosure on multiple subjects in public health emergencies, which will be the focus of a follow-up study.

## Conclusion

In this study, we examined the empirical information content published by government media on Weibo during the period of COVID-19. Research in this area broadens empirical theory's interpretation of the current practice of government information disclosure and identifies future research direction. First, in public health emergencies, government media's experience and information need to be in line with their media attributes. This should be based on three dimensions: social governance, medical expertise, and encouraging support in the fight against the epidemic. Second, the research illustrated a need for the Chinese government to improve its ability to harness new and past experiences to effectively contain public health emergencies. These are proposed to be important indicators to measure social governance. Experience and rational content should coexist and conflate in the disclosure of experience information and are appropriate to build a suitable system to manage public health emergencies. The content disclosed in public health system incidents is extremely important. Third, the government media disclosure of information and experiences about COVID-19 in digital format is evidence of the advances in policy formulation and implementation of digitalization and modernization of information disclosure. This is the outcome of social governance as outlined at the 19th Central Committee's Fourth Plenary Session. Modernization is also the connotation of the “four forces” of news and public opinion reported by General Secretary Xi Jinping at the 19th National Congress of the Communist Party of China (41). Finally, China's government media and other media's use of existing experience in information disclosure should be examined. This will help improve China's social governance capabilities in the information age toward a digital government. It will also provide an inexhaustible impetus for the future realization of phased headway from informatization to intelligence and intellect.

## Data Availability Statement

The original contributions presented in the study are included in the article/supplementary material, further inquiries can be directed to the corresponding author.

## Author Contributions

YZ proposed the content and direction of the research, contributed data and analysis tools, and wrote the paper. JS contributed data and analysis tools and wrote the paper. ZY analyzed the data and wrote the paper. All authors contributed to the article and approved the submitted version.

## Funding

The study was supported by the National Social Science Foundation project Study on the Poverty Alleviation Communication and Social Development of the Poverty-stricken Population in Old Revolutionary Base Areas (Grant No. 16BXW081).

## Conflict of Interest

The authors declare that the research was conducted in the absence of any commercial or financial relationships that could be construed as a potential conflict of interest.

## Publisher's Note

All claims expressed in this article are solely those of the authors and do not necessarily represent those of their affiliated organizations, or those of the publisher, the editors and the reviewers. Any product that may be evaluated in this article, or claim that may be made by its manufacturer, is not guaranteed or endorsed by the publisher.
